# Acoustic and flow data of fluidic and piezoelectric ultrasonic transducers

**DOI:** 10.1016/j.dib.2021.107280

**Published:** 2021-08-13

**Authors:** Benjamin Bühling, Stefan Maack, Eric Schönsee, Thorge Schweitzer, Christoph Strangfeld

**Affiliations:** a Bundesanstalt für Materialforschung und -prüfung (BAM), Unter den Eichen 87, Berlin 12205, Germany; b FDX Fluid Dynamix GmbH, Rohrdamm 88, Berlin 13629, Germany

**Keywords:** Ultrasound, Non-destructive testing, Air-coupled ultrasound, Fluidics, Acoustic-flow interaction, Piezoelectric transducer

## Abstract

This data article presents characteristic acoustic and flow data of a fluidic ultrasonic transducer as well as acoustic data of a commercial piezoelectric ultrasonic transducer used in non-destructive testing for civil engineering. The flow data has been acquired using hot-wire anemometry and a Pitot tube. The three-dimensional acoustic data of both devices has been acquired using a calibrated microphone. The distribution of characteristic acoustic properties of both transducers are extracted and given in addition to the raw data. The data presented in the article will be a valuable source for reference and validation, both for developing fluidic and alternate ultrasound generation technologies. Furthermore, they will give additional insight into the acoustic-flow interaction phenomena of high speed switching devices. This article is accompanying the paper Experimental Analysis of the Acoustic Field of an Ultrasonic Pulse Induced by a Fluidic Switch (Bühling et al., 2021) published in The Journal of the Acoustical Society of America, where the data is interpreted in detail and the rationale for characteristic sound properties of the fluidic transducer are given.

## Specifications Table

**Table:** 

Subject	Engineering
Specific subject area	Ultrasound generation
Type of data	Tables and time series
How data were acquired	Acoustic measurements: MV301 microphone system and MV302 amplifier (Microtech Gefell) Hot-wire measurements: 55P011 single-wire (Dantec), IFA-100 anemometer (TSI Inc.) Pitot tube measurements: custom bent tube with outer diameter 2mm and inner diameter 1.1mm), HDOB005 pressure sensor (First Sensor) Ambient conditions: WS 6750 weather station (Techno Line) Data Acquisition: USB-6361 (National Instruments) Software: Labview (National Instruments) for data acquisition, Python for processing
Data format	Raw data and processed data
Parameters for data collection	All data is acquired as a voltage. Microphone data is converted according to the manufacturer’s calibration, flow data is calibrated manually under consideration of atmospheric pressure, temperature and humidity
Description of data collection	The acoustic field and the mean flow field of a fluidic switch and the acoustic field of a piezoelectric air-coupled ultrasonic transducer were measured. The working fluid of the fluidic switch and the surroundings were air. The respective measurement probes were held constant while the fluidic switch was moved through several two-dimensional planes. For the acoustic measurement the whole switching cycle was acquired, for flow measurements the switch was operated in static mode.
Data source location	Institution: Federal Institute for Materials Research and Testing (BAM) City/Town/Region: Berlin Country: Germany
Data accessibility	Repository name: Harvard Dataverse Data identification number: https://doi.org/10.7910/DVN/OQYPC9[2]
Related research article	Benjamin Bühling, Christoph Strangfeld, Stefan Maack, and Thorge Schweitzer, “Experimental analysis of the acoustic field of an ultrasonic pulse induced by a fluidic switch”, The Journal of the Acoustical Society of America 149, 2150-2158 (2021) https://doi.org/10.1121/10.0003937[1]

## Value of the Data

• The data are results from detailed acoustic and flow measurements of a fluidic switch for ultrasonic pulse generation.
• This dataset is useful to researchers who are interested in acoustic-flow interaction and ultrasound generation.
• The data may be used to validate the operation conditions of fluidic ultrasonic transducers, compare novel ultrasound generation techniques and gain insight into acoustic-flow interaction.
• Reference data of the acoustic field of a commercial piezoelectric transducer is given, which is useful for future benchmarks of air-coupled ultrasonic transducers

## 1 Data Description

**Fig. 1:**
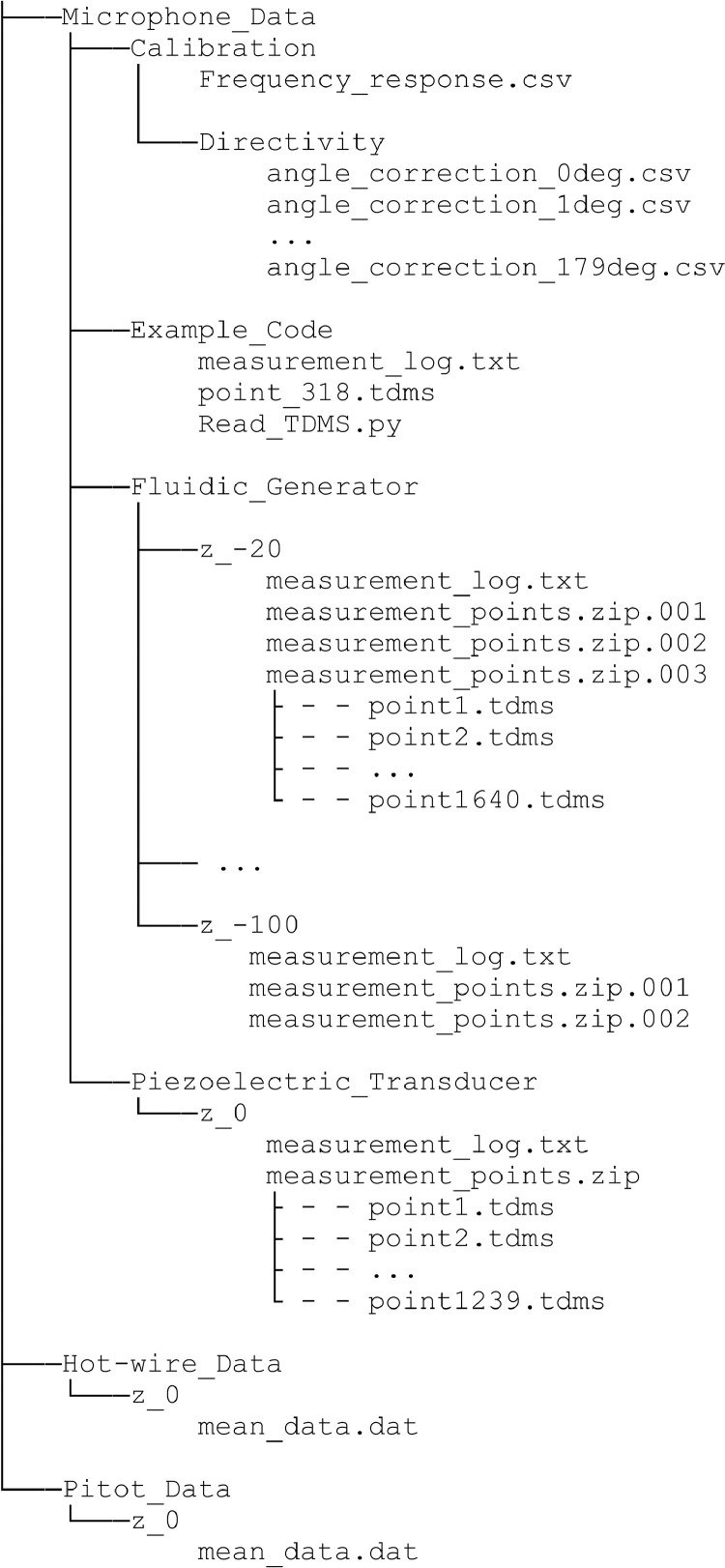
Data structure of the repository.

**Fig. 2:**
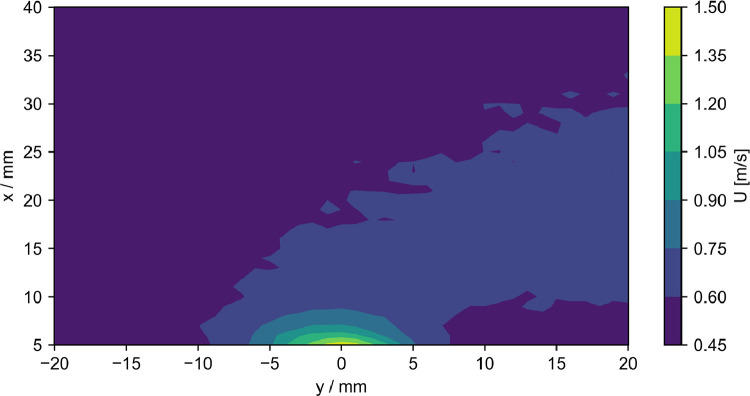
Mean velocity downstream of the fluidic transducer switched off, as measured with the hot-wire anemometer.

**Fig. 3:**
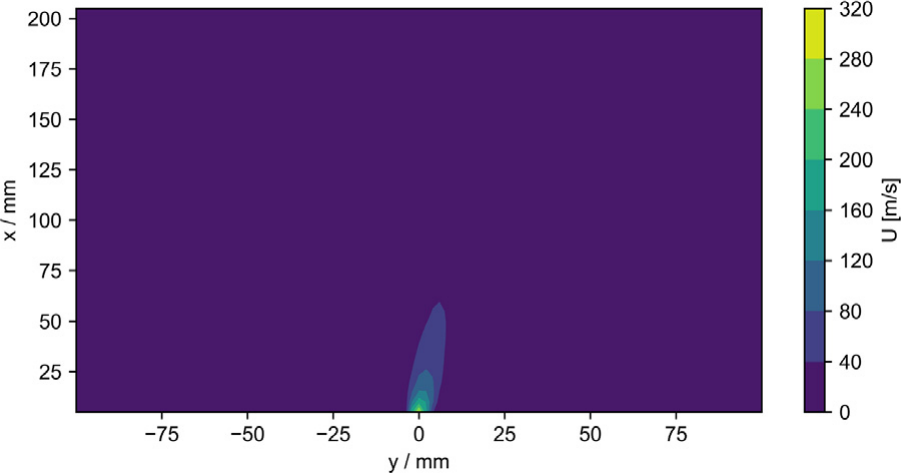
Mean velocity downstream of the fluidic transducer switched on, as measured with the Pitot tube.

**Fig. 4:**
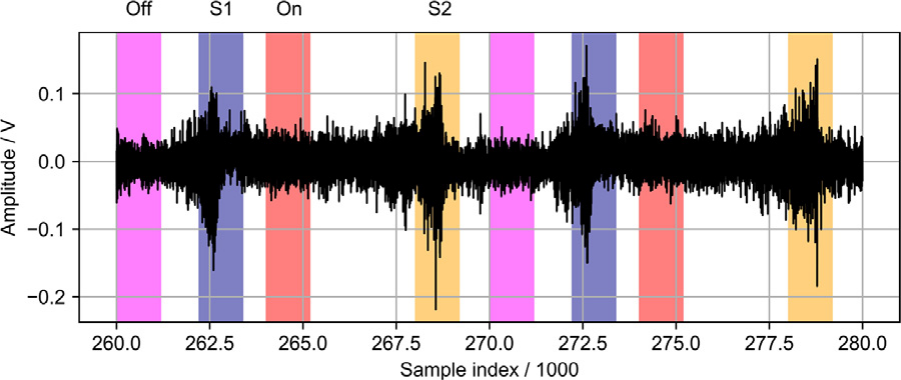
Section of the microphone data at (x,y,z)=(50,40,−20) mm. The section contains two switching periods, where the ranges to be processed are highlighted in color.

**Fig. 5:**
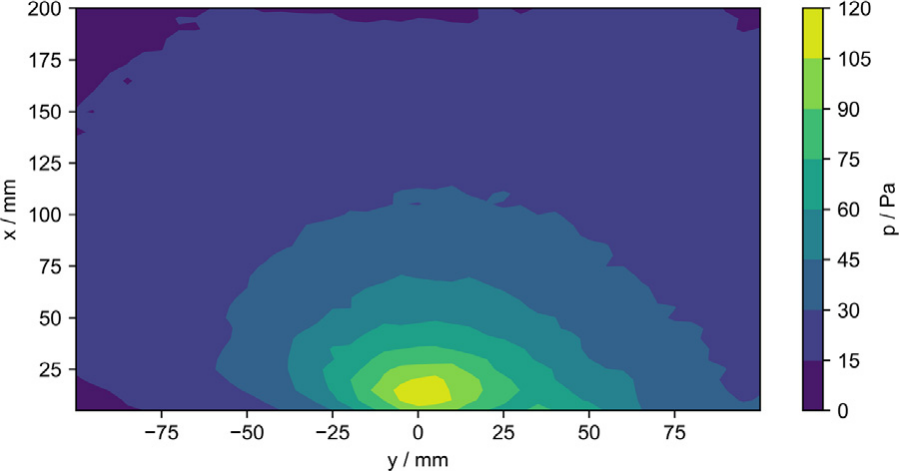
Median absolute pressure maximum, when switching on at z=−30 mm.

The data presented here contain acoustic and flow data downstream of the outlet of a fluidic ultrasonic transducer as well as the acoustic field of commercial piezoelectric air-coupled ultrasonic transducer. The acoustic data has been acquired using a calibrated microphone, the flow data using hot-wire anemometry and Pitot tube measurements. The dataset contains both raw and processed acoustic data as well as processed flow data. Additionally, calibration data for all microphone measurements and minimal workings examples for processing in Python are given. The file tree is shown in Fig. 1. The main directory is divided into the three types of measurements. Hot-wire and pitot tube measurement folders contain only one measurement plane orthogonal to the flow exit plane passing through the center of the flow outlet. The velocity data is given in.dat files along with the coordinates. A visualization of the data is given Figs. 2 and 3. The microphone measurement folder contains folders for both types of transducers. The fluidic transducer folder contains the 9 measurement planes in the range of z=−20,...,−100 mm. The piezoelectric transducer folder contains only the center plane at z=0 mm. All measurement plane folders contain.TDMS files of the voltage data obtained at one measurement point in the plane. The TDMS files also hold the sample rate and number of samples of the measurements. Additionally, a.txt file is given, that lists the cartesian coordinates of the measurement points and additional columns of processed data. The processing is described in the following section. Fig. 4 shows a section of time series of microphone data, where the regions to be processed are highlighted. An example for the median absolute pressure maximum in the z=−30 mm plane of the fluidic device is given in Fig. 5. The files needed for calibration are given in separate directory. Furthermore, the microphone measurement folder contains a minimum working example in Python for file-readout.

## 2 Experimental Design, Materials and Methods

**Fig. 6:**
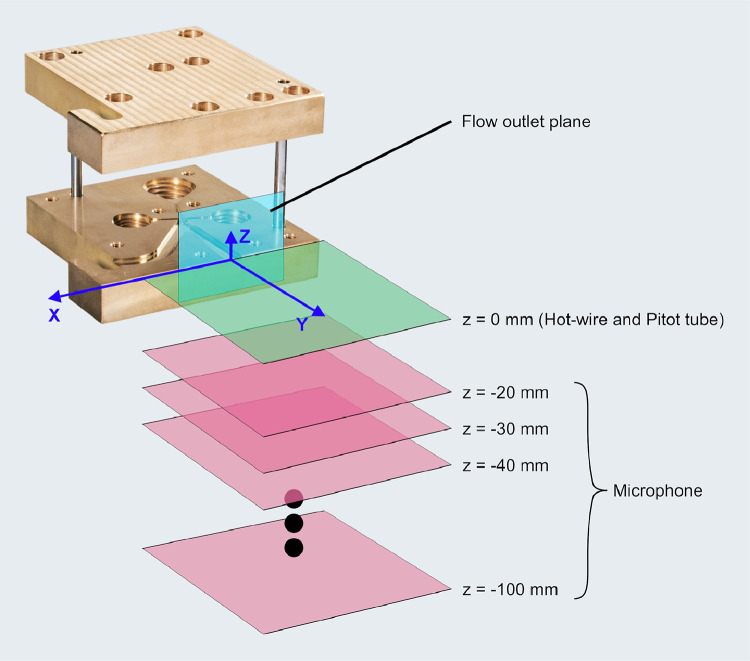
Measurement planes downstream of the fluidic ultrasonic transducer. For the piezoelectric transducer the flow outlet plane coincides with the actuation plane. The figure has been adapted from the accompanying study [1], with the measurement planes being added for this publication.

In order to measure the acoustic and flow fields of the fluidic ultrasonic transducer, this transducer was mounted to a two-dimensional stage, which was controlled by a stepper motor controller C 166-4 (isel Germany AG). The piezoelectric air-coupled ultrasonic transducer was mounted on the same system. The various probes were static while measuring a plane. To acquire microphone data from multiple planes, the microphone was moved manually in the third dimension. A schmeatic of the measurement area is shown in Fig. 6. All probes were directed perpendicular to the flow outlet plane.

### 2.1 Fluidic ultrasonic transducer operation

The fluidic ultrasonic transducer was connected to a constant source of pressurized air, where a pressure of 1.85 bar was applied to the supply port, 1.2 bar to the passive control port and 1.4 bar to the active control port. Further description of the transducer layout can be found in the accompanying study [1]. A Festo MHJ10 solenoid valve was used to control the active port. For pulse generation the solenoid valve was opened for 15 ms and a repetition rate of 25 Hz. At each measurement point 100 fluidic switching cycles were executed.

### 2.2 Piezoelectric transducer operation

The piezoelectric transducer NCG100-S63 (Ultran Group) was actuated with an 80 kHz rectangular pulse and a peak-to-peak voltage of 100 V. The repetition rate of the transducer was 50 Hz.

### 2.3 Microphone measurements

The microphone was directed toward the outlet plane fluidic transducer. Since an impingement of high velocity flow would damage the microphone, the closest plane measured was at a distance of z=20 mm from the flow outlet. The expanded uncertainty of the microphone in amplitude and frequency is 0.15 dB and 0.1 Hz, respectively.

### 2.4 Microphone data processing

**Fig. 7:**
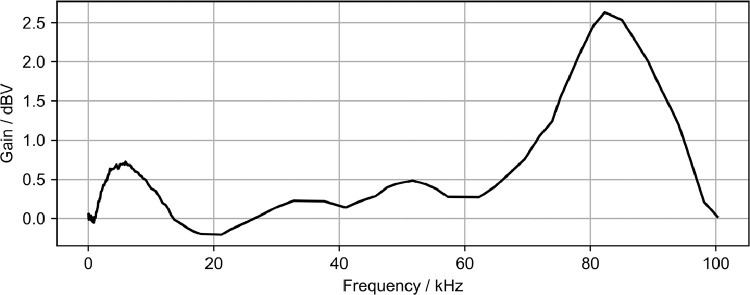
Microphone frequency response.

**Fig. 8:**
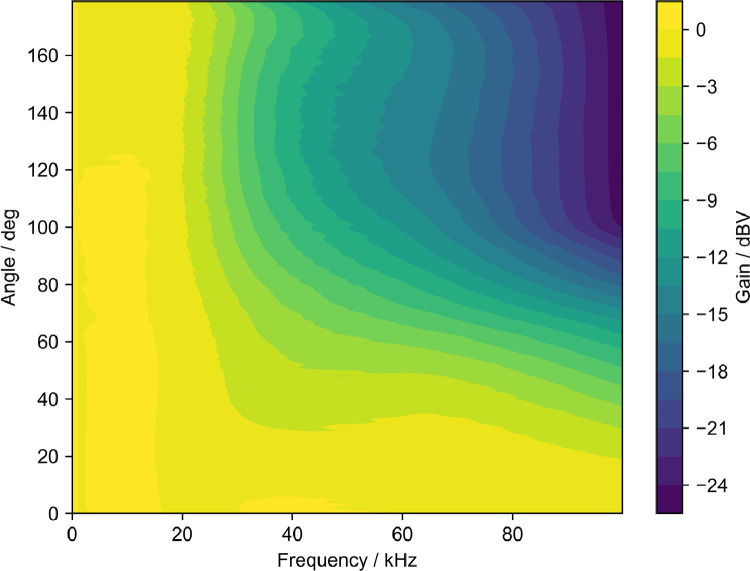
Interpolated microphone directivity.

The microphone data was corrected for the microphone frequency response and for the angular directivity of the microphone, which were supplied by the manufacturer and digitized using the WebPlotDigitizer software [5]. The respective curves are given in Figs. 7 and 8. To obtain only the ultrasonic sound pressure, a Butterworth low-pass filter was applied to the data. The time signal was then partitioned into 100 parts, one for each switching cycle. In each partition four time intervals of equal length were chosen, that corresponded to the stable states and switching intervals of each cycle, as outlined in the accompanying paper [1]. These time intervals are given in Table 1. In the time interval associated with the switching processes the maximum absolute sound pressure was extracted, in the time intervals of stable states, the standard deviation was calculated. This procedure conducted for every partition then averaged over the measurement point.

**Table 1:** Index range for extraction of switching events in all microphone measurements of the fluidic transducer. Abbreviations in brackets refer to Fig. 4.

Event	Index range
Switching on (S1)	2200:3400
Switching off (S2)	8000:9200
On	0:1200
Off	4000:5200

### 2.5 Pitot tube measurements

**Fig. 9:**
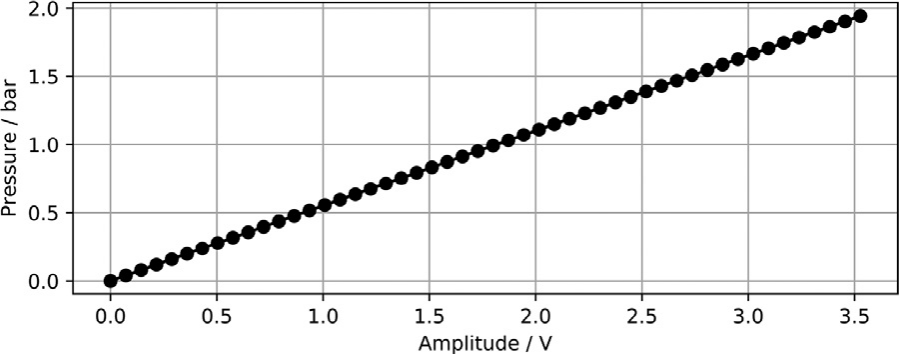
Calibration curve for pitot tube measurements.

The Pitot tube used to measure the jet flow of the fluidic transducer was custom built at Federal Institute for Materials Research and Testing. The differential pressure sensor was calibrated using the calibration curve given in Fig. 9. The tip of the probe was directed toward the outlet plane fluidic transducer and measurements were conducted during steady outflow from the main outlet of the fluidic device. The pressure signal was then converted to flow velocity using Bernoulli’s equation. The ambient air density was calculated from the Antoine equation [3] using ambient data readings from a weather station.

**Fig. 10:**
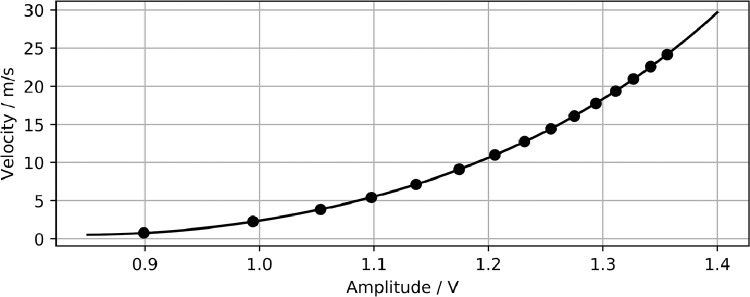
Calibration curve for hot-wire measurements.

### 2.6 Hot-wire measurements

The hot-wire measurements were conducted during steady suction flow of the fluidic transducer. The probe was inserted into the flow field parallel to the outlet plane in positive x direction. The wire was aligned with the z axis. The anemometer was calibrated in an open-return, suction wind tunnel at TU Berlin [4], following the calibration curve given in Fig. 10.

## CRediT Author Statement

**Benjamin Buhling:** Conceptualization, Methodology, Investigation, Software, Formal Analysis, Writing – original draft, Data Curation; **Stefan Maack:** Supervision, Project Administration, Writing – review & editing; **Eric Schonsee:** Investigation, Data Curation, Writing – Review & Editing; **Thorge Schweitzer:** Resources, Writing – Review & Editing; **Christoph Strangfeld:** Supervision, Project administration, Writing - review & editing.

## Declaration of Competing Interest

The Federal Institute for Materials Research and Testing (BAM), the employer of B. Bühling, C. Strangfeld, S. Maack and E. Schönsee, is holding a patent concerning air-coupled ultrasound generation using fluidic oscillators [6] of which C. Strangfeld and S. Maack are the inventors. T. Schweitzer is employed by FDX Fluid Dynamix GmbH, a company that develops and sells fluidic-based products.
